# Maladie rénale chronique: facteurs associés, étiologies, caractéristiques clinique et biologique à Lubumbashi en République Démocratique du Congo

**DOI:** 10.11604/pamj.2017.28.41.9810

**Published:** 2017-09-15

**Authors:** Serge Muleka Ngoie, Philippe Mulenga, Olivier Mukuku, Christian Ngama Kakisingi, Cédrick Milindi Sangwa, Pascal Tshimwang Nawej, Claude Mulumba Mwamba, Dophra Nkulu Ngoy, Faustin Wa Pa Manda Muteta

**Affiliations:** 1 Département de Médecine Interne, Faculté de Médecine, Université de Lubumbashi, République Démocratique du Congo; 2 Département de Santé Publique, Faculté de Médecine, Université de Lubumbashi, République Démocratique du Congo; 3 Département de Chirurgie, Faculté de Médecine, Université de Lubumbashi, République Démocratique du Congo; 4 Centre Médical du Centre-Ville (CMDC), Lubumbashi, République Démocratique du Congo

**Keywords:** Maladies rénales chroniques, facteurs associés, clinique, biologie, Lubumbashi, Chronic kidney disease, factors, clinical, biology, Lubumbashi

## Abstract

**Introduction:**

La maladie rénale chronique constitue un véritable problème mondial de santé publique du fait de l'augmentation de ses principaux facteurs de risque à savoir l'hypertension artérielle et le diabète sucré. Dans nos milieux à faible revenu et spécialement dans notre pays, peu d'études sont connues sur cette pathologie diagnostiquée à un stade très avancée et posant un problème de prise en charge.

**Méthodes:**

Il s'agit d'une étude descriptive transversale ayant été menée durant la période allant de juillet 2014 à juillet 2015 au service de dialyse de CMDC. Ont été inclus tous les patients avec taux de filtration glomérulaire inférieur à 60ml/min/1,73 m^2^ ou créatinine élevée au-delà de trois mois durant notre période d'étude L'objectif de cette étude est de décrire les caractéristiques sociodémographiques, les facteurs de risque et les paramètres biologiques de patients reçus pour insuffisance rénale.

**Résultats:**

Nous avons retenu 60 patients. L'âge moyen était de 51, 38+/-13, 47 ans avec la tranche d'âge la plus touchée comprise entre 50-59 ans. 51, 67% avaient un niveau d'instruction secondaire et 40% un niveau supérieur. Les facteurs de risque d'atteinte rénale étaient l' HTA 66, 64%, le diabète sucré 25%, l'usage des produits nephrotoxiques 35%, l'infection à VIH 11, 67%, l'obésité 10%, la drépanocytose 3, 3%. Le poids de naissance de naissance de nos patients ainsi que l'existence d'une maladie rénale familiale étaient des facteurs méconnus.85% de nos patients avaient un taux d'hémoglobine inférieur à 12g%.

**Conclusion:**

De cette observation, il ressort que l'âge de nos patients ne diffère pas de celui observé dans les autres milieux à revenu faible. Le niveau d'instruction de nos patients est plus élevé comparé aux autres études. Il serait mieux de développer des stratégies de dépistage précoce de la maladie rénale pour éviter d'aboutir à l'hémodialyse qui reste un traitement très onéreux.

## Abstract (en)

**Introduction:**

Chronic kidney disease is fast becoming a worldwide public health problem due to the increase of hypertension and diabetes mellitus, its main risk factors. In countries like DRC where majority of population are in the low income bracket, very few studies about this disease, usually diagnosed at a very advanced stage have been conducted. As a result of such, cases are not always properly taken care of and managed.

**Methods:**

We opted for a descriptive cross-sectional study and it was conducted during the period from July 2014 to July 2015 at CMDC dialysis service. Were included all patients with glomerular filtration rate lower than 60ml / min / 1,73 m2 or high level of creatinine longer than three months during the study period Goal. This study aims at describing the sociodemographic characteristics, risk factors and biological parameters of patients admitted for kidney failure.

**Results:**

We selected 60 patients. The average age was 51, 38 + / _ 13, 47 with the most affected included age group between 50-59 years. 51, 67% had completed secondary education and 40% higher. Risk factors of renal damage were the HTA 66, 64%, 25% diabetes mellitus, use of nephrotoxic products 35%, HIV infection 11, 67%, 10% obesity, sickle cell disease 3, 3%. The birth weight birth of our patients as well as existing renal disease in family were unknown factors. 85% of our patients had hemoglobin levels below 12 g%.

**Conclusion:**

From this observation, it appears that the age of our patients did not differ from that observed in other low-income communities. The level of education of our patients is higher compared to other studies. It would be better to develop strategies for early detection of kidney disease to avoid ending hemodialysis remains a very expensive treatment.

## Introduction

La maladie rénale chronique (MRC) constitue un véritable problème mondial de santé publique du fait de l'augmentation constante de ses taux d'incidence et de prévalence due à l'augmentation de ses principaux facteurs de risque à savoir le diabète sucré, l'hypertension artérielle, les maladies cardio- vasculaires, les collagénoses et le vieillissement de la population;et du coût élevé de sa prise en charge au stade terminal avec des résultats décevants [1]. Elle est définie par la persistance pendant plus de trois mois d'une diminution du débit de filtration glomérulaire(DFG)< 60ml/min/1,73m2 ,ou par un débit de filtration glomérulaire >60ml/min/1,73m^2^ associés à un ou plusieurs marqueurs d'atteinte rénale [2–4]. Les différentes études épidémiologiques menées à travers le monde estiment une prévalence variant de 10 -15% [5–13]. En Afrique Sub Saharienne celle-ci a été estimée à 13,9% [14]. Les projections de 2030 prévoient que plus de 70% de la population mondiale avec insuffisance rénale chronique terminale se retrouveront dans les pays en voie de développement dont fait partie la plupart des pays de l'Afrique Sub Saharienne. Il faudra noter qu'en 2004 uniquement 5% de cette population avait accès au traitement par suppléance rénale [15]. En République Démocratique du Congo, les études menées sur la prévalence de la maladie rénale dans la ville Province de Kinshasa l'ont estimée à 12,4% et 19,8% [16, 17]. Dans notre milieu, la seule étude portée à notre connaissance est celle qui a évalué les moyens d'accessibilité financière des patients insuffisants rénaux à l'hémodialyse ainsi que les facteurs de risque de mortalité de ces derniers. Il a été constaté que pour un cout moyen mensuel de 1270$ d'hémodialyse, 52,8% des patients avaient déclaré avoir un revenu mensuel de 205$ ; 34% 525$ et 13,2% 750$. Et que l'irrégularité de suivi en hémodialyse a été le facteur de risque de décès en hémodialyse soit 77% suivie de l'arrêt du traitement [18]. Face à cet état des choses, nous avons décidé d'apporter une pierre à la connaissancede la maladie rénale chronique à Lubumbashi. L'objectif général de cette étude sera de contribuer à l'amélioration de la prise en charge des patients insuffisants rénaux chroniques reçus pour la première fois au Centre Médical Du Centre-ville (CMDC) à l'Unité de Néphrologie/dialyse. Comme objectifs spécifiques : 1) : décrire les caractéristiques sociodémographiques ; 2) : décrire les facteurs associés et les étiologies de la maladie rénale chronique ; 3) : déterminer les paramètres cliniques et biologiques des patients avec maladie rénale chronique.

## Méthodes

**Type d'étude:** Il s'agit d'une étude descriptive transversale allant de la période de juillet 2014 à juillet 2015.

**Lieu d'étude:** Notre étude a été menée au Centre Médical du Centre-ville de Lubumbashi plus précisément à l'unité de Néphrologie/Dialyse qui est une institution privée la seule fonctionnelle dans notre ville ,deuxième de notre pays; existante depuis 2008 et équipée de 5 générateurs d'hémodialyse de marque Fresenius 4008B et organisant journellement 2 schifts matin et après-midi et recevant à peu près dix patients au quotidien.

**Population d'étude et critères d'inclusion:** Notre population d'étude a été constituée de tous les nouveaux patients de plus de 18 ans de race noir ayant fréquenté le service de néphrologie de CMDC durant notre période d'étude.

**Comme critères d'inclusion:** Tout patient de plus de 18 ans avec un dosage précédent de la créatininémie documenté à plus de 3 mois et/ou celui ayant bénéficié d'un suivi de créatininémie au-delà de 3 mois ; avec estimation du taux de débit de filtration glomérulaire≤ 60ml /min/1,73m^2^ selon la formule de Modification of Diet in RenalDisease (MDRD)[19].

**Critères de non inclusion:** Tout patient de moins de 18 ans ; tout patient avec MRC bénéficiant de l'hémodialyse ou ayant déjà été transplanté ; tout patient avec MRC avec grossesse.

### Variables d'étude

**Caractéristiques sociodémographiques:** Age défini par tranche; sexe: féminin ou masculin; niveau d'étude: primaire (≤6ans), secondaire (7-12 ans), supérieur (≥12ans) aucun (= 0 ans); facteurs associés reportés à l' interview: prise de tabac courante ou passée ;prise courante d' alcool ou passée; absence d' activité physique; histoire familiale du diabète sucré, de l'HTA, des maladies rénales; histoire personnelle de l'HTA, de diabète sucré des maladies cardiaques, des dyslipidémies, des maladies rénales, de goutte, drépanocytose, infection à HIV, de maladie systémique ;notion de prise des Anti Inflammatoires Non Stéroïdiens (AINS), des produits néphrotoxiques ,des produits traditionnelles ;notion d'infections urinaires à répétition [20].

**Les paramètres cliniques:** L'hypertension artérielle(HTA) définie par une PAS ≥140 mm Hg et/ou une PAD ≥90mmHg [21]; le diabète sucré définie par une glycémie à jeun ≥126 mg/dl [22]; l'indice de masse corporelle défini par le poids/taille au carré (kg/m^2^) : obésité ≥30; surpoids 25-29,9; normal 18,5-24,9; < 18,5 maigreur [23]; le syndrome métabolique a été défini sur base de la présence d'au moins trois critères suivant : HDL Cholestérol<35 mg/dl pour l'homme et 40 mg/dl pour la femme ; triglycérides >140mg/dl ; périmètre abdominal ≥102cm pour l'homme et ≥88 cm pour la femme glycémie élevée et une HTA [24]; la maladie rénale chronique a été classée selon KDIGO 2012 (Tableau 1) [2].

**Tableau 1:** Classification de la maladie rénale chronique

Stade	DFG (ml/min/1,73m^2^)	Description
**G1**	≥ 90	Maladie sans I Rénale
**G2**	60-89	I Rénale Chronique légère
**G3a**	45-59	I R chronique moyennement modérée
**G3b**	30-44	I R chronique moyennement modérée
**G4**	15-29	I R chronique sévère
**G5**	< 15	I R chronique Terminale

Nos paramètres biochimiques ont été mesurés à l'aide d'un spectrophotomètre de marque CYAN Start CY004 et hématologiques avec l'appareil CYAN hemato en respectant les procédures et valeurs de référence standard avec contrôle de qualité interne [25].

**Récolte des données:** la récolte des données s'est effectuée à l'aide d'une fiche d'enquête nous ayant permis de prélever les données sociodémographiques, cliniques et paracliniques

**Considérations éthiques:** Nous avons obtenu un consentement verbal de tous les patients. Ces derniers ont accepté la publication des résultats tout en protégeant leur identité.

**Analyses statistiques:** Nos données ont été traitées avec le logiciel Epi Info version 7.1. et Excel window 7. Nos variables quantitatives continues ont été présentées sous forme de moyenne avec écart type et les qualitatives en pourcentage. Pour la comparaison de moyennes et des proportions nous avons utilisé le test de T-student et le chi carré. Notre P value était considéré statistiquement significatif à un seuil < 0,05

## Résultats

**Paramètres sociodémographiques (** **Tableau 2** **):** L'âge moyen de nos patients était de 51,38±13,47 ans. 34 étaient de sexe masculin et 26 de sexe féminin. 51,67% avaient un niveau d'instruction secondaire, 45,00% supérieur et 3,33% primaire.

**Tableau 2:** Paramètres socio démographiques

Caractéristique	Effectif	Pourcentage
**Age**		
>30 ans	5	8,34%
30-39 ans	6	10,01%
40-49 ans	12	20,00%
50-59 ans	22	36,68%
60-69 ans	11	18,33%
≥70 ans	4	6,67%
**Sexe**		
Féminin	26	43 ,33%
Masculin	34	56,67%
**Niveau d’instruction**		
Primaire	2	3,33%
Secondaire	31	51,67%
Supérieur	27	45,00%

**Figure 1:**
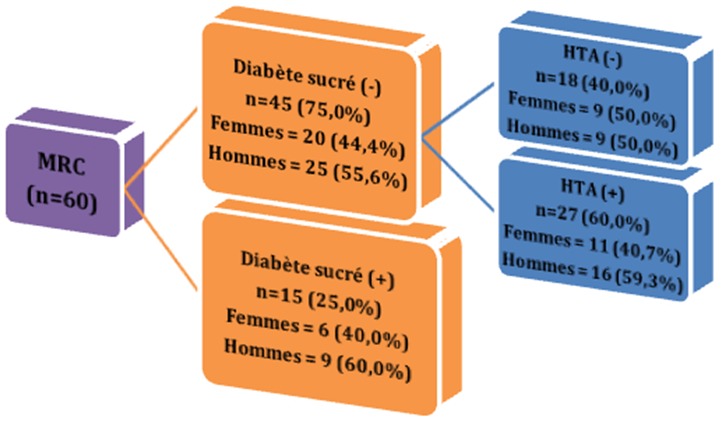
Répartition selon les paramètres cliniques

**Répartition des patients selon les facteurs associés (** **Tableau 3** **):** Il ressort des facteurs associés à la maladie rénale chronique que: 1) l'histoire familiale de l'HTA représente 46,67% et celle du diabète sucré 25%. 2) l'histoire personnelle des maladies rénales a été rapportée à 16% et celle des infections urinaires à répétition 10,00%. 3) la prise de tabac et d'alcool ont été des facteurs retrouvés chez les hommes que chez les femmes avec respectivement 26,47% et 64,71%; l'usage des AINS et des produits traditionnels ont été retrouvés respectivement à 36,67% et 35% dans les deux sexes. L'absence d'activité physique était notée 71,67% avec différence statistiquement significative dans les deux sexes. 4) 66,67% étaient hypertendus; 25% diabétiques; 11,67% infectés par le VIH; 5,0% avaient une maladie systémique; la goutte et la drépanocytose représentaient tous 3, 33%. 5) répartition des patients selon les étiologies (Figure 1).

**Tableau 3:** Répartition des patients selon les facteurs associés

Facteurs associés	Féminin (n=26)	Masculin (=34)	Total	P
Age≥60 ans	6(23,08%)	9(26,47%)	15(25%)	1,000
Histoire familiale d’HTA	11(42,31%)	17(50%)	28(46,67%)	0,7408
Histoire familiale de diabète sucré	4(15,38%)	11(32,35%)	15(25%)	0,2233
HTA	17(65,38%)	23(67,65%)	40(66,67%)	0,9266
Diabète sucré	6(23,08%)	9(26,47%)	15(25%)	1,000
Histoire personnelle de maladie rénale	3(11,54%)	7(20,59%)	10(16,67%)	0,4905
Prise d’alcool	4(15,38%)	22(64,71%)	26(43,33%)	0,0001
Prise de tabac	0(0%)	9(26,47%)	9(15%)	0,0037
Usage des AINS	8(30,77%)	14(41,18%)	22(36,67%)	0,5764
Usage de produits traditionnels	7(26,92%)	14(41,18%)	21(35%)	0,3821
Absence d’activité physique	24(92,31%)	19(55,88%)	43(71,67%)	0,0031
Notion de dyslipidémie	2(7,69%)	3(8,82%)	5(8,33%)	1,000
Histoire de maladies cardiaques	5(19,23%)	9(26,47%)	14(23,33%)	0,5550
Goutte	0(0%)	2(5,88%)	2(3,33%)	0,5005
HIV	3(11,54%)	4(11,76%)	7(11,67%)	1,000
Drépanocytose	1(3,85%)	1(2,94%)	2(3,33%)	1,000
Maladies systémiques	1(3,85%)	2(5,88%)	3(5,0%)	1,000
Infections urinaires à répétition	1(3,85%)	5(14,71%)	6(10,00%)	0,2206

Il a été noté que 25% des patients avec maladie rénale chronique étaient diabétiques et que du reste soit 75%, 60% étaient hypertendus et 40% avaient les autres causes.

**Répartition selon les paramètres cliniques (** **Tableau 4** **):** Il ressort de nos résultats que: 1) l'IMC moyen de nos patients était de 23,25±5,55kg/m^2^. On n'a pas noté de différence statistiquement significative dans les 2 sexes. 3) 10,0% des patients étaient obèses avec une différence statistiquement significative dans les deux sexes. 3) 66,67% des patients avaient des pressions artérielles élevées. 4) 13,33% avaient des chiffres glycémiques supérieurs à la normale. 5) 8,33% avaient un syndrome métabolique.

**Tableau 4:** Répartition selon les paramètres cliniques

Paramètres cliniques	Féminin	Masculin	Total	P
IMC moyen	24,51 ± 7,55	22,28 ± 3,11	23,25 ± 5,55	0,1245
Obésité	5 (19, 23%)	1 (2, 94%)	6 (10, 00%)	0,0492
Systole moyenne	158,38 ± 37,62	160,94 ±36,51	159,83 ±36,70	0,7918
Diastole moyenne	95,50 ± 22,49	93,97 ± 22,52	94,63 ± 22,33	0,7952
T.A élévée	17 (65, 38%)	23 (67, 65%)	40 (66, 67%)	0,9266
Glycémie	4 (15, 38%)	4 (11, 76 %)	8 (13, 33%)	0,7172
Glycémiemoyenne	112,84±50,51	109,38 ±53,09	110,88 ±53,09	0,8046
Syndrome métabolique	2 (7, 69%)	3 (8, 82%)	5 (8, 33%)	1,0000

**Répartition des paramètres biologiques selon les stades de la maladie: (** **Tableau 5** **)** Il ressort de nos résultats que: 1) la moyenne de l'acide urique sérique est de 6,85 ± 2,22 mg%. 2) l'hémoglobine moyenne est de 8,86 ±2,31 mg% avec une différence statistiquement significative en fonction du stade de la maladie. 3) la calcémie moyenne est de 8,71 ± 1,45mg%. 4) la kaliémie moyenne est de 4,65 ± 1,12 mg%.

**Tableau 5:** Répartition des paramètres biologiques selon les stades de la maladie

Variable	Stade <5(n=21)	Stade 5(n=39)	Total(n=60)	P
**Acide urique**				
Moyenne (±ET)	6,76 ± 2,37	6,90 ± 2,17	6,85 ± 2,22	0,8250
Hyperuricémie n (%)	12 (57, 14%)	17 (43, 59%)		0,4183
Hémoglobine				
Moyenne (±ET)	9,98 ± 2,15	8,26 ± 2,18	8,86 ±2,31	0,0047
Anémie n (%)	17 (80, 95%)	36 (92, 31%)		0,2262
**Calcémie**				
Moyenne (±ET)	8,54 ± 1,04	8,80 ± 1,64	8,71 ± 1,45	0,5211
Hypocalcémie n (%)	11 (52, 38%)	19 (48, 72%)		1,0000
**Potassium**				
Moyenne (±ET)	4,54 ± 1,12	4,71 ± 1,13	4,65 ± 1,12	0,5729
Hyperkaliémie n (%)	4 (19, 05%)	8 (20, 51%)		1,0000

## Discussion

De cette étude ayant porté sur les caractéristiques des patients reçus la première fois au service de Néphrologie-Dialyse de CMDC Lubumbashi pour maladie rénale chronique, nous avons récolté 60 patients dont l'âge moyen était de 51,38±13,47 ans avec un pic dans la tranche d'âge allant de 50-59 ans soit 36,68%. Ceci est similaire aux résultats obtenus dans les autres régions d'Afrique Sub Saharienne et autres pays en voie de développement comme au Maroc par Mohamed Reda El Faroukil et al. [26]; par Yaw Ampem et al. au Ghana [27]; Ulasi I et al. [28]; Shitu A O et al. [29] au Nigeria; Sakandé J et al. [30] au Burkina Faso; Sidi Mohamed Seck et al. [31] au Sénégal; Felix Burkhalter et al. [32] en Haïti et Sumaili E K [16, 17] à Kinshasa contrairement aux résultats obtenus dans les pays développés où la plupart des patients sont âgés de plus de 60 ans [5, 13]. Ceci s'explique par le fait que c'est la catégorie de la population socialement active qui s'expose facilement à certains facteurs environnementaux comme l'usage d'alcool; de tabac; des produits indigènes ainsi de plantes médicinales (super kabutshungu ; power…avec comme action fortifiant, aphrodisiaque…). L'HTA, l'une des principales causes de la maladie est aussi un élément touchant cette catégorie de la population due au stress social. Plus de la moitié de nos patients avaient un niveau d'instruction secondaire comparé à ceux des autres milieux [17, 26, 31, 32]; où on a trouvé que la plupart de ces patients ont un niveau d'étude inférieur à 6 ans. Dans notre cas ceci pourrait s'expliquer par le cadre dans lequel nous avons mené nos investigations qui est situé en pleine ville et privé limitant l'accès à la grande partie de la population, et qui nous fait supposer que la population ayant fréquenté cette institution n'est pas l'image réelle de notre société.

Des facteurs associés à la maladie, nous avons noté une prévalence non minime d'histoire familiale de l'HTA et de diabète sucré comme chez beaucoup d'auteurs [16, 31, 33, 34]; expliquée par le fait que ce sont les causes les plus importantes de la maladie [1, 5]; et par leur caractère héréditaire .La consommation d'alcool et de tabac ont été des facteurs beaucoup plus retrouvés chez l'homme avec une différence statistiquement significative pour les deux sexes, ceci s'expliquerait par nos habitudes culturelles qui empêchent les femmes à adopter ce style de vie. La prise des médicaments néphrotoxiques (AINS, antibiotiques,…), des produits traditionnels sont des facteurs incriminés dans notre milieu par la pratique de l'automédication, le manque de contrôle du secteur pharmaceutique par le pouvoir public. Ceci reste un phénomène observé dans plusieurs milieux en voie de développement [17, 26, 27] et l'absence d'activité physique comme facteur important dans notre étude s'explique le fait que notre population cherche une certaine aisance sociale en copiant aveuglement certaines mentalités (sédentarité, alimentation peu équilibrée, déplacement à moto ou en véhicule même pour des petites distances,…) ,et ignore la place du sport dans leur vie. Ce facteur empêcherait aussi la dégradation des protéines musculaires chez les insuffisants rénaux à la base d'une augmentation du taux de la créatinine [35].

L'HTA et le diabète sucré ont représenté plus de 50% comme dans la plupart d'études menées en Afrique Sub Saharienne et ailleurs [15] avec respectivement 45% et 25%. Ceci est dû par une augmentation du changement comportemental dans notre milieu où la population s'adonne à un régime alimentaire copié à l'occident et peu contrôlé (excès de sucre, de sel, des graisses polysaturées…); et autres habitudes comme le tabac, l'alcool,….Il existe aussi un faible contrôle et faible suivi thérapeutique de la plupart de cette catégorie de patients qui disparaissent dans la nature ou qui vont se faire traiter chez les tradipraticiens. L'obésité a été retrouvée chez 10% de nos patients. Ce facteur a été retrouvé aussi dans les autres études menées dans nos pays à faible revenu, expliqué la mauvaise hygiène alimentaire et le manque d'activité physique. Et ceci a été beaucoup plus observé chez les femmes avec une différence statistiquement significative. Chez ces dernières nous constatons de plus en plus dans notre milieu qu'elles sont véhiculées et elles vivent dans une situation dans laquelle elles sont sédentaires. L'infection à VIH a été rapportée à 11,67% comparée à d'autres études qui ont trouvé sa prévalence à 12% par Sumaili et al, 4% Ampem et al, 3,1% Felix Burkhalter et al. Nous pourrons dire que ces résultats reflètent la grande diminution de la fréquence des glomérulonéphrites infectieuses jadis incriminées comme cause de la maladie rénale chronique avec l'usage des antis microbiens (ARV, antibiotiques, antiparasitaires,…). Il faudra signaler que la prévalence de la maladie rénale chronique chez les patients infectés par le VIH en Afrique Sub Saharienne était estimée entre 6-45% [15]. Les autres néphropathies n'ont pas pu être évaluées par manque d'investigations très poussées. La grande difficulté dans notre milieu reste le manque d'un néphrologue formé. Les biopsies rénales n'étaient pas faites et certains malades décidaient d'aller à l'étranger pour aller poursuivre le reste de la prise en charge. Ceci reste un grand défi à relever.

Pour ce qui est du stade de la maladie au moment de la première consultation néphrologique, nous avons noté dans notre série que 65% étaient au stade terminal et 33,3% stade 4. Ces résultats sont identiques à ceux observés dans la plupart des pays en voie de développement [25, 26, 31, 32]. Contrairement à ceux observés dans les pays développés et émergents [5, 11, 13].)où moins de 50% sont vus au stade supérieur à 3. Ceci du fait de la faible politique de dépistage et du fait que la plupart des médecins généralistes ne sont pas bien informés sur la pathologie. Le manque de moyen financier et d'assurance médicale fait que les patients ne consultent pas régulièrement surtout la catégorie des patients avec pathologies chroniques comme le diabète sucré ,l'HTA et autres qui préfèrent se fier aux autres alternatives telles la prière, l'usage des plantes traditionnelles. Sur le plan des paramètres biologiques, nous avons trouvé la valeur de l'hémoglobine moyenne à 8,86±2,31g% chez plus de 85% des patients témoignant d'une anémie qui reste une grande complication de la maladie comme dans la plupart d'études [25, 26, 36, 37] et avions constaté que celle-ci diminue au fur et à mesure que la maladie évolue comparé aux autres paramètres. Il faut noter que celle-ci n'est pas toujours très bien investiguée du fait qu'il existe une grande variabilité des comorbidités associées à la maladie rénale chronique [38]. Pour ce qui est des autres paramètres tels que la kaliémie, la calcémie, l'uricémie qui étaient dans les normes, nous pourront accuser l'usage de certains produits comme les diurétiques, les inhibiteurs de l'enzyme de conversion,…..qui probablement peuvent influer sur le taux sérique de ceux-ci soit en les diminuant ou en les augmentant. Nous aurions bien voulu présenter les autres paramètres liés aux complications tels que le pH, le bicarbonate, la parathormone; ceci n'étaient pas possible par la limite du laboratoire à effectuer ces examens au début de notre étude.

## Conclusion

Dans notre milieu, la maladie rénale chronique atteint le sujet adulte socialement actif et avec un niveau d'instruction moyen. Elle est principalement due à l'HTA, le diabète sucré. Chez l'homme elle est associée à la prise d'alcool, de tabac et des plantes traditionnelles. Chez les femmes, elle semble associer à l'obésité. Sur le plan clinique et biologique, nous avons noté que nos patients avaient une hémoglobine moyenne inférieure à la normale. Des complications paracliniques, nous avons noté l'anémie qui était associée au stade évolutif de la maladie.

### Etat des connaissances actuelles sur le sujet

- C'est un véritable problème de santé publique dans le monde du fait l'augmentation de son incidence;
- Elle touche plus les personnes âgées de plus de 65 ans, hypertendus, diabétiques;
- Son dépistage précoce permet de ralentir son évolution vers le stade ultime d'insuffisance rénale chronique terminale nécessissant le traitement par suppléance rénale qui reste couteux.

### Contribution de notre étude à la connaissance

- Cette étude donne un aperçu de la maladie dans notre milieu;
- Elle vient renforcer les observations faites dans les autres milieux à revenu faible;
- De nos observations pourront naitre des perspectives pour des études futures.

## Conflits d’intérêts

Les auteurs ne déclarent aucun conflit d'intérêts.
